# Development of Simple Analytical Method for B-Group Vitamins in Nutritional Products: Enzymatic Digestion and UPLC-MS/MS Quantification

**DOI:** 10.1155/2021/5526882

**Published:** 2021-05-05

**Authors:** Quang Huy Nguyen, Anh Quoc Hoang, Thi My Hanh Truong, Thi Diu Dinh, Thi Thuy Le, Thi Huyen Trang Luu, Viet Chien Dinh, Thi Minh Thu Nguyen, Thi Trang Vu, Thi Anh Huong Nguyen

**Affiliations:** ^1^Faculty of Chemistry, University of Science, Vietnam National University, 19 Le Thanh Tong, Hanoi 10000, Vietnam; ^2^Department of Pharmaceutics and Pharmaceutical Technology, Faculty of Pharmacy, Thai Nguyen University of Medicine and Pharmacy, Thai Nguyen 24000, Vietnam; ^3^National Institute for Food Control (NIFC), 65 Pham Than Duat, Hanoi 10000, Vietnam

## Abstract

A method for the simultaneous determination of seven B-group vitamers including thiamine, riboflavin, nicotinamide, niacin, pyridoxine, pyridoxal, and pyridoxamine in nutritional products by using enzymatic digestion followed by LC-MS/MS quantification was studied. The LC-MS/MS conditions such as MS transitions, mobile phase programs, and ammonium formate buffer concentrations, and sample treatment procedures (e.g., concentrations of buffer solution, digestion temperature, and digestion time) were investigated. The analytical method performance was evaluated by multiple criteria such as selectivity, linearity, detection and quantification limits, repeatability, reproducibility, and recovery by using real sample matrices. The validated method was successfully applied to analyze vitamin B concentrations in different nutritional products like ultra-heat-treated milk, powdered milk, and nutritional powder. Vitamin B concentrations varied over a wide range from lower than detection limits to about 9000 *µ*g/100 g, depending on vitamin groups, compound forms, and sample types. The measured concentrations of B-group vitamins in our samples were generally in good agreement with values of label claims.

## 1. Introduction

The B-group vitamins comprise eight types of water-soluble vitamins (i.e., vitamin B1, B2, B3, B5, B6, B7, B9, and B12) and one type can exist in different forms, also called vitamers [1]. Vitamins in general and B-group vitamins in particular are essential for maintaining various body functions in humans, and vitamin deficiencies may cause adverse health effects [1, 2]. Because foods are the most important vitamin sources, a nutritious and balanced diet can eliminate deficiencies [1–3]. However, negative impacts of vitamin overdose have also been noted [4]. The World Health Organization has tabulated recommended vitamin amounts in human nutrition [2]. The development of a reliable and effective method for the simultaneous determination of multiple vitamin types and forms in food and nutritional products is needed to characterize the occurrence of such nutrients in food and to provide useful information for regulatory agencies, manufacturers, and consumers [5, 6].

In order to extract B-group vitamins from food matrices, several extraction and purification methods were applied, e.g., solid-phase extraction (SPE) with C18 adsorbent [7, 8], dispersive SPE with molecularly imprinted biopolymers [9], treatment with precipitation reagents [5, 10], and extraction with acetonitrile [6, 11]. Each method has its own advantages and limitations. From a green chemistry point of view, the use of organic solvents [6, 11], heavy metal salts [5], and potentially toxic chemicals like trichloroacetic acid [10] should be avoided. In addition, strong acid hydrolysis may be responsible for the appearance of some impurities, which interfere with chromatographic signals of some B-group vitamins [5]. The application of enzymatic digestion to extract B-group vitamins from complex food matrices rich in proteins and carbohydrates has been reported by various studies [12, 13]. As for instrumental analysis of B-group vitamins, liquid chromatography with mass spectrometry detection (especially ultra-performance liquid chromatography-tandem mass spectrometry UPLC-MS/MS) has become the standard method due to its outstanding specificity, sensitivity, and efficiency [12–14].

In the present study, four types with seven forms of B-group vitamins were examined, including vitamin B1 (thiamine), B2 (riboflavin), B3 (nicotinamide and niacin), and B6 (pyridoxine, pyridoxal, and pyridoxamine). We focused on three nutritional products such as ultra-heat-treated milk, powdered milk, and nutritional powder, which are usually fortified with micronutrients including B-group vitamins. The samples were treated by a simple and “green” extraction method, which utilized enzymatic digestion without the use of organic solvents and heavy metal salts. Analytical method performance (i.e., UPLC-MS/MS with isotope dilution/internal standard quantification) was strictly validated, showing adequate specificity and accuracy. Concentrations of individual vitamers over a wide range (from *µ*g to mg per 100 g) in nutritional products were reported with comprehensive insights into their total levels, profiles, and validity of nutrition food labels. To our knowledge, this is among the first studies to investigate the occurrence of multiple B-group vitamers in nutritional products in Vietnam as well as Southeast Asia.

## 2. Materials and Methods

### 2.1. Standards and Reagents

Analytical standards including thiamine hydrochloride (THI), riboflavin (RIB), nicotinamide (NIC), niacin (NIA), pyridoxine hydrochloride (PYN), pyridoxal hydrochloride (PYL), and pyridoxamine dihydrochloride (PYM), and other chemicals and reagents (e.g., ammonium formate, formic acid, methanol, acid phosphatase, papain, and *α*-amylase) were purchased from Sigma-Aldrich (St. Louis, MO, USA). Isotope-labelled internal standards (^13^C_4_-THI, ^13^C_4_^15^N_2_-RIB, ^2^H_4_-NIC, ^2^H_4_-NIA, ^13^C_4_-PYN, ^2^H_3_-PYL, and ^2^H_3_-PYM) were obtained from IsoSciences (Ambler, PA, USA). Double-distilled deionized water was used to prepare buffer and standard solutions. The enzyme cocktail solution including 200 ± 10 mg of acid phosphatase, 80 ± 5 mg of *α*-amylase, and 400 ± 10 mg of papain was prepared in 200 mL of 50 mM ammonium formate solution and adjusted to pH 4.0–4.5 with formic acid. The native mixed working standard (MWS) and internal standard stock mixtures (ISSM) were prepared in 50 mM ammonium formate solution. The working standard solutions (WS1 to WS7) were prepared at individual concentrations of native compounds ranging from 0.20 to 1200 ng/mL and internal standard concentrations ranging from 0.20 to 20 ng/mL.

### 2.2. Instrumentation and Optimization of LC-MS/MS Conditions

In this study, a liquid chromatograph (ACQUITY UPLC H-Class; Waters) equipped with a tandem mass spectrometer (Xevo TQD; Waters) and an ACQUITY UPLC BEH C18 column (100 mm × 2.1 mm × 1.7 *μ*m, 130 Å; Waters) was used. Basic parameters of the LC-MS/MS system in this study are summarized in Table 1.

Based on the basic parameters shown in Table 1 with mobile phase A as 20 mM ammonium formate solution referred from the AOAC Official Method 2015.14 [14], we performed three experiments to subsequently determine the following: (1) MS transitions of target compounds and internal standards; (2) effect of mobile phase programs on retention times and separation efficiency; and (3) effect of ammonium formate concentration in mobile phase A on signal intensities of the analytes. The MS transition parameters (e.g., quantitative and qualitative ions, cone voltage, and collision energy) were automatically optimized. Different programs of the mobile phase were investigated, including one isocratic program and three gradient programs (Table 2). Concentrations of ammonium formate for optimization ranged from 5 to 50 mM.

### 2.3. Optimization of Sample Preparation Procedure

Optimization experiments for the sample preparation procedure were performed by using a nutritional powder sample. Extraction conditions showing highest recovered amounts of vitamins in this sample were selected. We investigated the effects of (1) concentrations of ammonium formate in a range of 5 to 100 mM, (2) digestion temperature in a range of 35 to 50°C, and (3) digestion time in a range of 3 to 16 h, on extraction efficiency of 7 vitamins. The nutritional powder sample (10 ± 0.3 g) was reconstituted in distilled water (total weight 100 ± 2 g) by using a magnetic stirrer. An aliquot of 1 g reconstituted sample was transferred to a 50 mL tube, spiked with internal standards (100 *µ*L of ISSM standard) and 5 mL of the enzyme cocktail solution, and mixed by using a vortex mixer. The sample tube was then incubated in a thermostatic shaker overnight. The extract was transferred to a 25 mL volumetric flask, made up to 25 mL with 50 mM ammonium formate solution, and filtered through a 0.2 *µ*m PTFE membrane before LC-MS/MS analysis.

### 2.4. Method Validation

The specificity/selectivity and linearity of quantification for seven vitamers were confirmed according to the criteria proposed by the AOAC International [14] and the Commission Decision 2002/657/EC of the European Communities [15]. Based on the optimized procedure, additional nutritional product samples were analyzed to evaluate method performance. Three types of sample matrices were examined including ultra-heat-treated (UHT) milk, powdered milk, and nutritional powder. For each sample type, repeatability (within-day replicate analysis of a real sample, *n* = 6), reproducibility (between-days replicate analysis of a real sample, *n* = 4), method detection and quantification limits (replicate analysis of a low-concentration sample, *n* = 10), and recovery (replicate analysis of matrix-spiked samples at three spiking levels, *n* = 3 for each level) were evaluated. The accuracy of our method was also confirmed by method development and analytical results of infant formula samples from the AOAC's interlaboratory study (in 2018) and the Nestlé's internal tests (in 2019 and 2020).

### 2.5. Application of the Validated Method to Nutritional Products

The validated analytical method was applied to determine concentrations of 7 B-group vitamers in different nutritional products including UHT milk (*n* = 5), powdered milk (*n* = 5), and nutritional powder (*n* = 5) samples. All the samples were obtained randomly from several dealerships and groceries in Hanoi City, Vietnam, between 2019 and 2020.

## 3. Results and Discussion

### 3.1. MS/MS Conditions

The MS/MS conditions including mass transitions, cone voltage, and collision energy of seven vitamers and respective internal standards were automatically optimized and the results are shown in Table 3. The mass transitions of the target compounds in our study are in good agreement with those reported by the AOAC Official Method 2015.14 [14]. For each compound, one quantitative ion (product ion) and two qualitative ions (precursor ion and one product ion) were assigned. This scheme is in accordance with performance criteria of LC-MS/MS proposed by the Commission Decision 2002/657/EC, showing 4 identification points [15]. The MS/MS conditions tabulated in Tables 1 and 3 were used for next experiments.

### 3.2. LC Conditions

#### 3.2.1. Mobile Phase Programs

With mobile phase A as 20 mM ammonium formate solution and mobile phase B as methanol, we analyzed the working standard WS5 (individual concentrations ranging from 6 to 600 ng/mL) under four programs listed in Table 2. Retention times of the target compounds are shown in Table 4 and chromatograms are presented in Figure S1 of Supplementary data. In the isocratic program, all the vitamers were eluted within 2.5 min and some compounds were almost coeluted (e.g., NIC, PYL, and PYN). In addition, peaks of native and internal standards of NIA and PYM were not sharp and balanced (i.e., tailing peaks). A suitable gradient program is therefore necessary. The program Gradients 1 and 2 gave tailing peaks for NIA and PYM. The program Gradient 3 was selected because it provided well-resolved, sharp, and balanced peaks of all the compounds.

#### 3.2.2. Effect of Ammonium Formate Concentrations in Mobile Phase

**Figure 1:**
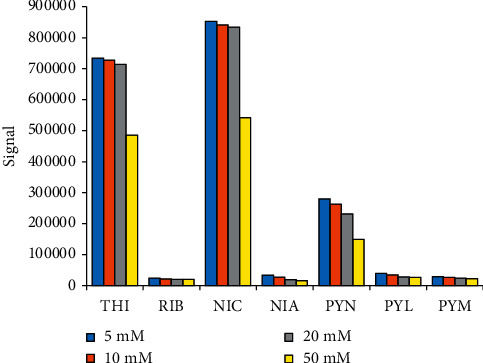
Effect of ammonium formate concentrations on signals of B-group vitamins in the working standard WS5.

The mobile phase A with concentrations of ammonium formate of 5, 10, 20, and 50 mM was investigated. Signal intensities of seven vitamers in the working standard WS5 are presented in Figure 1. In general, signals decreased with the increasing salt concentrations. The intensities were not significantly different with salt concentrations from 5 to 20 mM, which were much higher than those from 50 mM. With salt concentration of 5 mM, signals of RIB, PYM, and PYL were not balanced with shoulder or tailing peaks. Therefore, we selected mobile phase A as 10 mM ammonium formate solution. Some other studies used 20 mM ammonium formate [14, 16], but concentration of 10 mM was also applied elsewhere [17]. Finally, the mobile phases A (10 mM ammonium formate) and B (methanol) were used with program Gradient 3 (Table 2). Total runtime was 10 min.

### 3.3. Sample Preparation Procedure

#### 3.3.1. Effect of Ammonium Formate Concentrations

**Figure 2:**
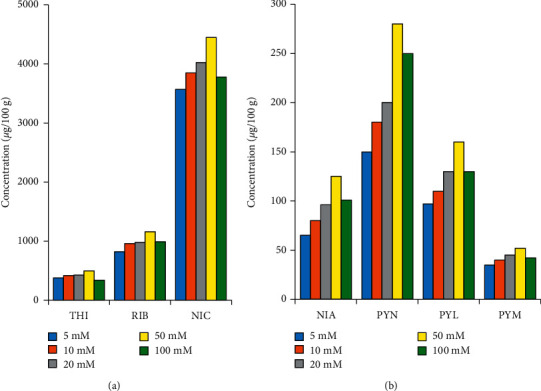
Effect of ammonium formate concentrations on extraction efficiency of B-group vitamins in nutritional powder matrix.

The test samples (about 1 g of reconstituted nutrition powder) were spiked with internal standards and digested with 5 mL of enzyme cocktail at 37°C for 12 h. Then, the digested mixtures were transferred to 25 mL volumetric flasks and diluted with ammonium formate solutions at concentrations of 5, 10, 20, 50, and 100 mM. Concentrations of seven vitamers in the test samples with different ammonium formate solutions are presented in Figure 2. Accordingly, the highest extraction efficiency of all compounds was obtained at salt concentration of 50 mM. This solution (i.e., 50 mM ammonium formate) was also used by the AOAC Official Method 2015.14 [14].

#### 3.3.2. Effect of Digestion Temperature

**Figure 3:**
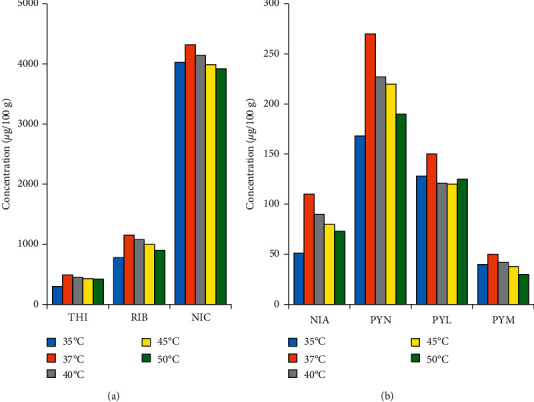
Effect of digestion temperature on extraction efficiency of B-group vitamins in nutritional powder matrix.

The test samples (about 1 g of reconstituted nutrition powder) were spiked with internal standards and digested with 5 mL of enzyme cocktail solution at for 12 h at different temperatures as 35, 37, 40, 45, and 50°C. Then, the digested mixtures were transferred to 25 mL volumetric flasks and diluted with 50 mM ammonium format solution. Concentrations of seven vitamers in the test samples with different digestion temperatures are shown in Figure 3. It is obvious that the sample treated at 37°C has the highest concentrations of all compounds. From 40°C onwards, amounts of vitamins recovered from the matrix decreased with the increasing temperature. Among B-group vitamins, vitamin B1 (THI) is relatively sensitive to high temperature as compared to others (e.g., vitamin B2 and B6) [18]. However, the temperature range investigated in this study did not exceed 50°C and is still lower than normal water temperature used to prepare formula (e.g., about 70°C) [19]. In addition, concentrations of THI did not change significantly over the temperature range from 37 to 50°C (RSD = 7%), suggesting that vitamin stability is not likely to be an important factor. Therefore, the extraction efficiency was largely attributed to enzyme activities, which were strongly affected by incubation temperature. The optimum temperatures of papain, *α*-amylase, and acid phosphatase were 65, 37, and 37°C, respectively [20–22]. As a result, digestion temperature higher than 37°C may inhibit enzymatic activity of *α*-amylase and acid phosphatase, which prevents complete liberation of free vitamins. Based on the optimization results, digestion temperature of 37°C was selected.

#### 3.3.3. Effect of Digestion Time

**Figure 4:**
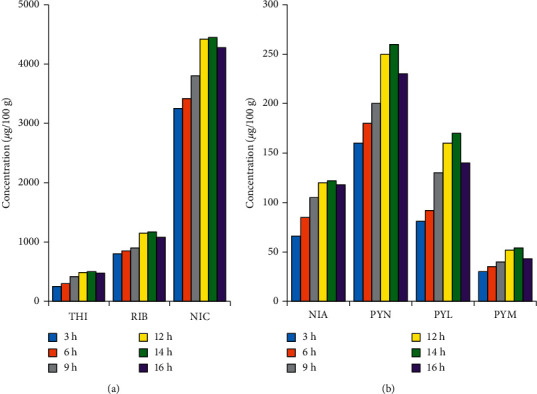
Effect of digestion time on extraction efficiency of B-group vitamins in nutritional powder matrix.

The test samples (about 1 g of reconstituted nutrition powder) were spiked with internal standards and digested with 5 mL of enzyme cocktail solution at 37°C over different periods as 3, 6, 9, 12, 14, and 16 h. As shown in Figure 4, extracted amounts of vitamins from the matrix gradually increased from 3 h to 14 h and then decreased after 16 h digestion. Concentrations of most compounds obtained after 12 h and 14 h digestion were not significantly different. Therefore, the optimal digestion time varied between 12 h and 14 h. As compared to other sample preparation methods (e.g., protein precipitation and acid hydrolysis), operation time of the enzymatic digestion method is much longer [12]. In our laboratory, the samples were incubated overnight, which helps to save time during working hours. Furthermore, accuracy is minimally dependent on small changes in digestion time by working near the peak of the signal/digestion time relationship.

#### 3.3.4. Optimal Sample Preparation Procedure

Before analysis, the samples (i.e., UTH milk, powdered milk, and nutritional powder) were homogenized thoroughly. The powdered milk or nutritional powder sample (10 ± 0.3 g) was reconstituted in distilled water (total weight 100 ± 2 g) by using a magnetic stirrer. An aliquot of 1 g UHT milk or reconstituted powder sample was transferred to a 50 mL tube, spiked with internal standards and 5 mL of the enzyme cocktail solution, and mixed by using a vortex mixer. The sample tube was then incubated at 37°C in a thermostatic shaker overnight (about 12 to 14 h). The extract was transferred to a 25 mL volumetric flask, filled up to 25 mL with 50 mM ammonium formate solution, and filtered through a 0.2 *µ*m PTFE membrane before LC-MS/MS analysis.

### 3.4. Method Validation Results

#### 3.4.1. Method Specificity/Selectivity

As described above, the specificity and selectivity of our analytical method meet the requirements of the AOAC International (i.e., each compound was confirmed by one precursor ion and two product ions) [14] and the Commission Decision 2002/657/EC (i.e., 4 identification points) [15]. Procedural blank samples (*n* = 10) were analyzed together with test and real samples, showing no signal of any target compound. Possible contamination from chemicals, reagents, and laboratory ware and environment was excluded. Results of the standards, samples, and matrix-spiked samples also indicated good agreement between signals (i.e., retention time and mass transitions) of all compounds from standards and from samples. In addition, the isotope dilution/internal standard method for quantification utilizing stable isotope-labelled compounds has been demonstrated as the most appropriate technique for quality control in LC-MS/MS. In general, our quantification method is specific and selective for the determination of multiple B-group vitamin forms in food matrix. Representative chromatograms of vitamers and internal standards in each sample type are shown in Figure S2, indicating distinct and clear appearance of these compounds in sample matrices.

#### 3.4.2. Calibration Curves

We prepared seven working standard solutions (WS1 to WS7) to construct calibration curves for all the target compounds. Concentration ranges of seven vitamers in the calibration standards were as follows: THI (2.7–136), RIB (11.5–230), NIC (23–1170), NIA (2.4–120), PYN (3.0–149), PYL (0.23–11.6), and PYM (0.26–13.2) ng/mL. The working standard solutions were prepared similar to real samples, including digestion with 5 mL of the enzyme cocktail solution, incubation at 37°C for about 12 h, constitution in 25 mL of 50 mM ammonium formate solution, and filtration through a 0.2 *µ*m PTFE membrane before LC-MS/MS analysis. Parameters of the calibration curves of seven vitamers are presented in Table 5, including equations, coefficients of determination, working ranges, and biases. The coefficients of determination of all compounds were higher than 0.998 within the respective working ranges. The biases (relative errors from known concentrations) were less than ±8% for all compounds, which meet the requirements of the AOAC International [14].

#### 3.4.3. Method Detection Limits, Quantification Limits, and Quantification Ranges

Based on screening results, we selected real samples with low vitamin concentrations (i.e., individual concentrations ranging from 1.70 ± 0.10 for PYL to 11.9 ± 1.0 *µ*g/100 g for NIC) and performed replicate analysis (*n* = 10) to determine method detection limits (MDLs) and quantification limits (MQLs). The MDLs and MQLs were estimated as three times and ten times of standard deviations, respectively. As shown in Table 6, MDLs of seven vitamers ranged from 0.28 to 2.8 *µ*g/100 g for liquid samples and from 2.8 to 28 *µ*g/100 g for powder samples, showing high sensitivity of our method. The quantification ranges span over three orders of magnitude, indicating that the method can be applied for samples with wide vitamin concentration ranges (i.e., *µ*g to mg per 100 g).

#### 3.4.4. Method Repeatability, Reproducibility, and Recovery

The repeatability (within-day replicate analysis of real samples, RSD_r_) and reproducibility (between-days replicate analysis of real samples, RSD_R_) of our method were determined for three sample types including UHT milk, powdered milk, and nutritional powder. There is no significant difference in the RSD_r_ (2.9–9.1%) and RSD_R_ (2.1–9.8%) values of individual vitamers between the three sample types (Table 7). The RSD values of our method were lower than 10%, indicating adequate precision. Our RSD values also satisfy the criteria proposed by the AOAC International for RSD_r_ < 15% and RSD_R_ < 22% at a concentration level of 100 ppb [23]. Representative samples of the three matrices were spiked with known amounts of vitamin standards at three concentration levels and analyzed to determine method recovery. The spiking levels varied between compounds and sample types, spanning over a wide range from 50 to 5000 *µ*g/100 g. The recoveries of all target compounds ranged from 80 to 107%, showing good accuracy. These recovery values fall within the acceptable range (80 to 110%) expected by the AOAC International for concentration levels of 100 ppb to 10 ppm [23]. The reliability of our method was also confirmed by method development results of infant formula samples from the AOAC's interlaboratory study in 2018 [14]. In addition, we also joined the Nestlé's internal tests in 2019 and 2020 for infant formula samples. Ratios of vitamin B1, B2, B3, and B6 concentrations measured by our method and the assigned values ranged from 79 to 113% with z-score values ≤2, suggesting acceptable levels of accuracy.

### 3.5. Concentrations of B-Group Vitamins in Nutritional Products

The validated method was applied to analyze concentrations of seven B-group vitamers in nutritional products obtained from dealerships and groceries in Hanoi City, Vietnam. The analytical results of real samples are tabulated in Table 8. THI, RIB, NIC, and PYN were quantified in all the samples. NIA was detected in milk samples (5/5 powdered milk samples and 3/5 UHT milk samples) but not in nutritional powder samples. Two other forms of vitamin B6 (i.e., PYL and PYM) were found in powdered milk samples only. Concentrations of NIC (724–8630 *µ*g/100 g) were generally higher than the remaining compounds (not detected–2480 *µ*g/100 g). Vitamin B concentrations in the powder samples (i.e., powdered milk and nutritional powder) were higher than liquid samples (i.e., UHT milk). However, these concentrations were derived for original samples and “actual” concentrations in prepared forms of the powder samples were not considered. Total vitamin B concentrations (i.e., vitamins B1, B2, B3, and B6) measured by our method were compared with label values, showing good agreement with average measured/labelled ratio as 96 ± 11%.

For the contributions of vitamers in total vitamin concentrations, our results indicate that NIC and PYN were the principal components of vitamin B3 and B6, respectively. NIC accounted for 95 to 100% (99 ± 1%) of total vitamin B3. Nutrition information of vitamin B3 on the product labels is usually reported as “vitamin B3” or “niacin,” although NIA was found at low levels or even not detected. Our findings were in good agreement with those reported for infant formula products [24, 25]. These observations suggest the need for manufacturer responsibility to report exact nutrition information in their product labels. NIC was also more abundant than NIA in human milk [26] and fresh cow, goat, and buffalo milk [27]. In our samples, PYN accounted for 90 to 100% (98 ± 4%) of total vitamin B6. In the powdered milk samples, proportions of PYN, PYL, and PYM in total vitamin B6 were 93 ± 3%, 4 ± 2%, and 2 ± 1%. Significant levels of PYL were found in infant formula samples (22–26% of PYN + PYL) [14]. PYL was major vitamer found in human milk (87–97% of PYN + PYL + PYM) [26]. Besides, significant percentages of PYL and some other vitamin B6 forms (e.g., pyridoxal 5′-phosphate and 4-pyridoxic acid) were detected in fresh milk samples [27]. Therefore, detailed and comprehensive analysis of different vitamin forms, rather than total vitamins and major vitamers, is needed in future studies.

## 4. Conclusions

In this study, a simple and solvent-free analytical method for the extraction of B-group vitamins in nutritional products was studied. Liquid samples (including UHT milk and reconstituted milk and nutritional powder) were digested with enzyme mixtures, diluted by ammonium formate solution, and filtered before quantification by using LC-MS/MS method. Our optimized method shows adequate specificity, accuracy, repeatability, reproducibility, and sensitivity for the simultaneous determination of seven B-group vitamers at a wide concentration range up to three orders of magnitude (e.g., *µ*g to mg per 100 g). The method was successfully applied to measure vitamin B concentrations in several nutritional products purchased from Hanoi City, Vietnam, showing agreement between measured and labelled values of total vitamin levels. However, the discrepancies resulting from vitamer recognition and contribution (i.e., for vitamins B3 and B6) should be taken into account. Our results suggest that detailed characterization of vitamin forms in food and nutritional products is necessary.

## Figures and Tables

**Table 1:** Basic parameters of the LC-MS/MS system in this study.

Parameter	Mode and setting value
Mobile phase A	Ammonium formate in water
Mobile phase B	Methanol
Flow rate	0.15 mL/min
Injection volume	10 *µ*L
Column temperature	30°C
Ionization	Electrospray ionization in positive mode (ESI+)
Capillary voltage	2500 V
Desolvation gas temperature	500°C
Desolvation gas flow	800 L/h
Cone gas flow	150 L/h
Collision gas flow	0.15 mL/min
Nebulizer	7 bar
Acquisition	Multiple reaction monitoring (MRM)

**Table 2:** Investigation of mobile phase programs for the determination of B-group vitamins in this study.

Program	Time (min)	%A	%B
Isocratic	0–5.0	50	50

Gradient 1	0–0.5	99	1
	2.5	92	8
	5.0	10	90
	6.0	10	90
	6.1–10.0	99	1

Gradient 2	0–0.5	92	8
	2.5	80	20
	3.0	50	50
	6.0	10	90
	7.0–10.0	95	5

Gradient 3	0–0.1	99	1
	0.5	95	5
	2.5	92	8
	4.0–5.0	10	90
	6.0–10.0	99	1

**Table 3:** MS/MS parameters for the determination of B-group vitamins in this study (bold numbers indicate quantitative ions).

Compound	Precursor ion (*m*/*z*)	Product ion (*m*/*z*)	Cone voltage (V)	Collision energy (eV)
THI	265	81	20	30
		122	24	34

^13^C_4_-THI	269	81	24	28
		122	22	16

RIB	377	198	34	38
		243	34	22

13C_4_^15^N_2_-RIB	383	175	44	38
		249	44	22

NIC	123	80	44	16
		96	44	18

^2^H_4_-NIC	127	84	44	18
		100	44	18

NIA	124	80	40	20
		106	40	20

^2^H_4_-NIA	128	84	44	16
		109	44	16

PYN	170	134	30	20
		152	30	13

^13^C_4_-PYN	174	138	28	20
		156	28	12

PYL	168	94	24	22
		150	24	12

^2^H_3_-PYL	171	97	22	24
		153	22	12

PYM	169	134	24	20
		152	24	12

^2^H_3_-PYM	172	136	26	20
		155	26	14

**Table 4:** Retention times (min) of B-group vitamins obtained by different mobile phase programs.

Compound	Isocratic	Gradient 1	Gradient 2	Gradient 3
THI	2.02	5.21	4.54	5.14
RIB	2.47	5.77	4.99	5.79
NIC	2.14	5.34	4.60	5.27
NIA	2.09	3.54	2.56	3.36
PYN	2.15	5.22	4.45	4.97
PYL	2.14	4.71	3.72	4.26
PYM	1.93	2.76	1.81	2.74

**Table 5:** Calibration curves of B-group vitamins.

Compound	Calibration curve equation (*A*: ratio of native/internal standard signal, *C*: concentration ng/mL)	Coefficient of determination (*R*^2^)	Working range (ng/mL)	Bias (%)
THI	*A* = 1.196 × *C* + 0.752	0.9993	3–130	−5.6–5.7
RIB	*A* = 0.937 × *C* + 4.670	0.9990	10–200	−6.3–7.2
NIC	*A* = 1.825 × *C* + 6.585	0.9995	30–1200	−3.9–5.4
NIA	*A* = 1.178 × *C* + 0.959	0.9984	3–120	−3.9–5.5
PYN	*A* = 1.634 × *C* + 0.520	0.9992	3–140	−4.2–5.0
PYL	*A* = 3.200 × *C* + 0.385	0.9992	0.3–10	−3.6–4.0
PYM	*A* = 12.122 × *C* + 0.752	0.9994	0.3–10	−2.6–4.9

**Table 6:** MDLs, MQLs, and quantification ranges (*µ*g/100 g) of B-group vitamins in nutritional products.

Compound	Liquid samples		Powder samples		Quantification range
	MDL	MQL	MDL	MQL	
THI	2.4	8.1	24	81	10–2000
RIB	2.3	7.8	23	78	10–4000
NIC	2.8	9.5	28	95	10–15,000
NIA	1.9	6.4	19	64	10–4000
PYN	2.7	9.2	27	92	10–2000
PYL	0.28	0.95	2.8	9.5	2–2000
PYM	0.35	1.2	3.5	12	2–2000

**Table 7:** Repeatability, reproducibility, and recovery of B-group vitamins in nutritional products.

Compound	Parameter	UHT milk	Powdered milk	Nutritional powder
THI	RSD_r_ (%)	3.2	4.8	4.3
	RSD_R_ (%)	5.7	5.8	5.1
	Recovery (%)	80–105	86–105	82–103

RIB	RSD_r_ (%)	4.7	4.5	4.5
	RSD_R_ (%)	9.7	7.2	7.1
	Recovery (%)	91–102	89–105	90–107

NIC	RSD_r_ (%)	2.9	3.8	4.3
	RSD_R_ (%)	6.0	5.5	2.1
	Recovery (%)	82–107	90–105	85–95

NIA	RSD_r_ (%)	7.1	5.0	3.3
	RSD_R_ (%)	8.1	7.4	6.6
	Recovery (%)	85–104	91–98	86–89

PYN	RSD_r_ (%)	5.6	3.2	7.0
	RSD_R_ (%)	9.8	5.8	9.5
	Recovery (%)	93–101	87–103	94–102

PYL	RSD_r_ (%)	7.3	8.5	9.1
	RSD_R_ (%)	8.2	6.1	9.1
	Recovery (%)	98–105	93–106	91–96

PYM	RSD_r_ (%)	6.9	4.3	4.2
	RSD_R_ (%)	3.5	5.1	5.4
	Recovery (%)	100–105	85–99	90–96

**Table 8:** Concentrations (*µ*g/100 g) of B-group vitamins in nutritional products purchased in Hanoi, Vietnam (UM: UHT milk, PM: powdered milk, NP: nutritional powder, ND: not detected).

	THI	RIB	NIC	NIA	PYN	PYL	PYM
UM-1	23.8	100	1130	11.4	81.9	ND	ND
UM-2	93.7	110	1170	14.8	86.0	ND	ND
UM-3	78.1	122	1050	48.6	98.0	ND	ND
UM-4	23.8	91.0	933	ND	86.0	ND	ND
UM-5	37.1	90.0	724	ND	80.0	ND	ND
PM-1	460	780	8630	65.0	559	40.5	20.8
PM-2	2080	2480	7310	110	2300	10.0	41.0
PM-3	1050	1130	7540	90.0	910	45.0	24.0
PM-4	1160	1240	6560	76.0	1120	52.0	21.0
PM-5	570	1100	6650	69.0	701	35.8	23.6
NP-1	590	620	1720	ND	160	ND	ND
NP-2	420	450	4250	ND	120	ND	ND
NP-3	630	590	1730	ND	110	ND	ND
NP-4	420	120	1200	ND	130	ND	ND
NP-5	710	110	6350	ND	280	ND	ND

## Data Availability

The main part of the research data is included within the article. Other data can be made available from the corresponding author upon request.

## Supplementary Materials

Supplementary Materials Figure S1: chromatograms of B-group vitamins and internal standards in standard solutions by different mobile phase programs. Figure S2: chromatograms of B-group vitamins and internal standards in nutritional products (Supplementary Materials).

## References

[1] LeBlanc J. G., LeBlanc J. G., Giori G. S. D. (2018). Introductory chapter: B-group vitamins. *B Group Vitamins Current Uses and Perspectives*.

[2] World Health Organization (WHO) (2004). *Vitamin and Mineral Requirements in Human Nutrition*.

[3] Munsell H. E. (1940). Vitamins and their occurrence in foods. *The Milbank Memorial Fund Quarterly*.

[4] Dhyani A., Chander V., Singh N. (2019). Overdose risk of vitamins: a review. *Journal of Pharmaceutical and Scientific Innovation*.

[5] Zafra-Gómez A., Garballo A., Morales J. C., García-Ayuso L. E. (2006). Simultaneous determination of eight water-soluble vitamins in supplemented foods by liquid chromatography. *Journal of Agricultural and Food Chemistry*.

[6] Suh J. H., Yang D. H., Lee B. K. (2011). Simultaneous determination of B group vitamins in supplemented food products by high performance liquid chromatography-diode array detection. *Bulletin of the Korean Chemical Society*.

[7] Moreno P., Salvadó V. (2000). Determination of eight water- and fat-soluble vitamins in multi-vitamin pharmaceutical formulations by high-performance liquid chromatography. *Journal of Chromatography A*.

[8] Gentili A., Caretti F., D’Ascenzo G. (2008). Simultaneous determination of water-soluble vitamins in selected food matrices by liquid chromatography/electrospray ionization tandem mass spectrometry. *Rapid Communications in Mass Spectrometry*.

[9] Ostovan A., Ghaedi M., Arabi M. (2018). Hydrophilic multitemplate molecularly imprinted biopolymers based on a green synthesis strategy for determination of B-family vitamins. *ACS Applied Materials & Interfaces*.

[10] Albalá-Hurtado S., Veciana-Nogués M. T., Izquierdo-Pulido M., Mariné-Font A. (1997). Determination of water-soluble vitamins in infant milk by high-performance liquid chromatography. *Journal of Chromatography A*.

[11] Bachmann T., Maurer A., Rychlik M. (2020). Development of a LC-MS/MS method using stable isotope dilution for the quantification of individual B6 vitamers in fruits, vegetables, and cereals. *Analytical and Bioanalytical Chemistry*.

[12] Fatima Z., Jin X., Zou Y. (2019). Recent trends in analytical methods for water-soluble vitamins. *Journal of Chromatography A*.

[13] Noh M. F. M., Gunasegavan R. D. N., Khalid N. M. (2020). Recent techniques in nutrient analysis for food composition database. *Molecules*.

[14] McClure S. (2020). Simultaneous determination of total vitamins B_1_, B_2_, B_3_, and B_6_ in infant formula and related nutritionals enzymatic digestion and LC-MS/MS—a multi-laboratory testing study final action: AOAC Official method 2015.14. *Journal of AOAC International*.

[15] The Commission of the European Communities (2002). Commission decision of 12 august 2002 implementing council directive 96/23/EC concerning the performance of analytical methods and the interpretation of results (2002/657/EC). *Official Journal of the European Communities*.

[16] Byrd N. (2012). *Rapid, Sensitive and Cost-Effective Detection of B Vitamins in Foods by UHPLC/MS/MS*.

[17] Goh E. (2010). *Rapid Analysis of Water-Soluble Vitamins in Infant Formula by Standard-Addition*.

[18] Riaz M. N., Asif M., Ali R. (2009). Stability of vitamins during extrusion. *Critical Reviews in Food Science and Nutrition*.

[19] Department of Food Safety (2007). *How to Prepare Formula for Bottle-Feeding at Home*.

[20] Payne C. T., Tarté R. (2009). “Chapter 8 enzymes. *Ingredients in Meat Products: Properties, Functionality and Applications*.

[21] Divakaran D., Chandran A., Chandran R P. (2011). Comparative study on production of *α*-Amylase from *Bacillus licheniformis* strains. *Brazilian Journal of Microbiology*.

[22] Mobley D. M., Chengappa M. M., Kadel W. L., Stuart J. G. (1984). “Effect of pH, temperature and media on acid and alkaline phosphatase activity in “clinical””and “nonclinical” isolates of *Bordetella bronchiseptica*. *Canadian Journal of Comparative Medicine*.

[23] AOAC Official Methods of Analysis (2016). *Appendix F: Guidelines for Standard Method Performance Requirements*.

[24] Viñas P., López-Erroz C., Balsalobre N., Hernández-Córdoba M. (2003). Reversed-phase liquid chromatography on an amide stationary phase for the determination of the B group vitamins in baby foods. *Journal of Chromatography A*.

[25] Reuter W. M., Reddy S., Dalmia A. (2016). *Analysis of Water-Soluble Vitamins in Infant Formulaby UHPLC-MS/MS*.

[26] Ren X. N., Yin S. A., Yang Z. Y. (2015). Application of UPLC-MS/MS method for analyzing B-vitamins in human milk. *Biomedical and Environmental Sciences*.

[27] Shetty S. A., Young M. F., Taneja S., Rangiah K. (2020). Quantification of B-vitamins from different fresh milk samples using ultra-high performance liquid chromatography mass spectrometry/selected reaction monitoring methods. *Journal of Chromatography A*.

