# Supplementary material for: Development of Simple Analytical Method for B-Group Vitamins in Nutritional Products: Enzymatic Digestion and UPLC-MS/MS Quantification
Source: J Anal Methods Chem. 2021 May 5;2021:5526882. doi: 10.1155/2021/5526882 (PMC8116160; doi:10.1155/2021/5526882)
Supplement: Supplementary Materials — Figure S1: chromatograms of B-group vitamins and internal standards in standard solutions by different mobile phase programs. Figure S2: chromatograms of B-group vitamins and internal standards in nutritional products (Supplementary Materials). [file 5526882.f1.docx]

# Journal of Analytical Methods in Chemistry

# Development of Simple Analytical Method for B-Group Vitamins in Nutritional Products: Enzymatic Digestion and UPLC-MS/MS Quantification

Quang Huy Nguyen,^1,2^ Anh Quoc Hoang,^1^ Thi My Hanh Truong,^1^ Thi Diu Dinh,^1^ Thi Thuy Le,^3^ Thi Huyen Trang Luu,^3^ Viet Chien Dinh,^3^ Thi Minh Thu Nguyen,^1^ Thi Trang Vu,^3*^ and Thi Anh Huong Nguyen^1*^

^1^ Faculty of Chemistry, University of Science, Vietnam National University, Hanoi, 19 Le Thanh Tong, Hanoi 10000, Vietnam.
^2^ Department of Pharmaceutics and Pharmaceutical Technology, Faculty of Pharmacy, Thai Nguyen University of Medicine and Pharmacy, Thai Nguyen 24000, Vietnam.

^3^ National Institute for Food Control (NIFC), 65 Pham Than Duat, Hanoi 10000, Vietnam.

Correspondence should be addressed to: Thi Anh Huong Nguyen ([nguyenthianhhuong@hus.edu.vn](mailto:nguyenthianhhuong@hus.edu.vn)) Thi Trang Vu ([trangvt@nifc.gov.vn](mailto:trangvt@nifc.gov.vn))

## Supplementary data

**Fig. S1.** Chromatograms of B-group vitamins and internal standards in standard solutions by different mobile phase programs

**Fig. S2.** Chromatograms of B-group vitamins and internal standards in nutritional products

**Fig. S1.** Chromatograms of B-group vitamins and internal standards in standard solutions by different mobile phase programs

**Isocratic program**

|  |  |
| --- | --- |
|  |  |
|  |  |
|  |  |
|  |  |
|  |  |
|  |  |

**Gradient 1**

|  |  |
| --- | --- |
|  |  |
|  |  |
|  |  |
|  |  |
|  |  |
|  |  |

**Gradient 2**

|  |  |
| --- | --- |
|  |  |
|  |  |
|  |  |
|  |  |
|  |  |
|  |  |

**Gradient 3**

|  |  |
| --- | --- |
|  |  |
|  |  |
|  |  |
|  |  |
|  |  |
|  |  |

**Fig. S2.** Chromatograms of B-group vitamins and internal standards in nutritional products

**Powdered milk**

|  |  |
| --- | --- |
|  |  |
|  |  |
|  |  |
|  |  |
|  |  |
|  |  |

**UHT milk**

|  |  |
| --- | --- |
|  |  |
|  |  |
|  |  |
|  |  |
|  |  |
|  |  |

**Nutritional powder**

|  |  |
| --- | --- |
|  |  |
|  |  |
|  |  |
|  |  |
|  |  |
|  |  |
